# Platypnea-Orthodeoxia: A case of PFO with aortic compression

**DOI:** 10.1007/s12471-025-01943-6

**Published:** 2025-03-21

**Authors:** Joana Guimarães, Patrícia Costa, Joana Ferreira

**Affiliations:** 1 Cardiology Department, Coimbra Hospital and University Centre (CHUC), Coimbra, Portugal; 2 Intensive Care Department, Coimbra Hospital and University Centre (CHUC), Coimbra, Portugal

A 59-year-old previously healthy woman developed severe hypoxaemia during a mastectomy, with postoperative oxygen saturation levels as low as 77%, preventing ventilator weaning. Once extubated in the ICU, her oxygen saturation varied significantly (78–97%) despite high-flow nasal oxygen therapy. Hypoxaemia worsened in upright positions and improved in the right lateral decubitus position. No hypoxaemia had been noted before this episode.

Pulmonary computed tomography angiography revealed a 50-mm ascending aortic aneurysm with no evidence of pulmonary embolism. A transoesophageal echocardiogram showed the aneurysm compressing the right atrium, distorting the atrial septum, and *stretching* a patent foramen ovale (PFO) (Fig. 1a), causing a significant right-to-left shunt (Fig. 1b; see Supplementary Material, video 1). This confirmed platypnoea-orthodeoxia syndrome (POS).

**Fig. 1:**
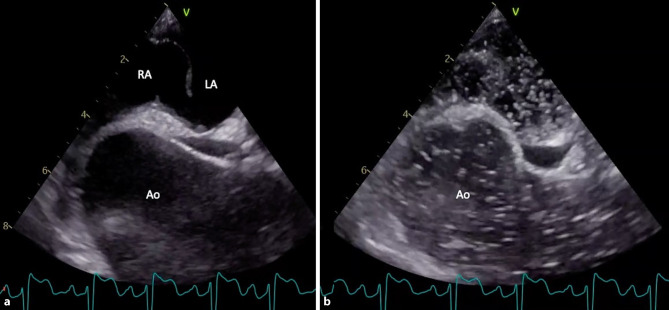
Ascending aortic aneurysm, compressing the right atrium and stretching a patent foramen ovale (**a**); large right-to-left shunt visualised on bubble study (**b**)

We hypothesise that mechanical ventilation during surgery increased right cardiac chamber pressures, unmasking hypoxaemia. Percutaneous PFO closure normalised oxygen saturation to 99% on room air. The aneurysm was newly diagnosed during this episode, and the patient remains under cardiology follow-up to monitor its progression.

## Supplementary Information

Video showing hypoxemia with postural changes

